# Feasibility of Single-Lead Apple Watch Electrocardiogram in Atrial Fibrillation Detection Among Heart Failure Patients

**DOI:** 10.1016/j.jacadv.2024.101051

**Published:** 2024-07-03

**Authors:** Rachel H. Heo, Farid Foroutan, Enza De Luca, Lisa Albertini, Valeria E. Rac, Joseph A. Cafazzo, Steve G. Hershman, Juan G. Duero Posada, Heather J. Ross, Yasbanoo Moayedi

**Affiliations:** a University of Toronto, Toronto, Canada; b Ted Rogers Centre for Heart Research, University of Toronto, Toronto, Canada; c Program for Health System and Technology Evaluation, Toronto General Hospital Research, Toronto, Canada; d Institute, University Health Network, Toronto, Canada; e Institute of Health Policy, Management and Evaluation, Dalla Lana School of Public Health, University of Toronto, Toronto, Canada; f Centre for Digital Therapeutics, Biomedical Engineering, University Health Network, Toronto, Canada; g Institute of Health Policy, Management and Evaluation, Institute of Biomedical Engineering, University of Toronto, Toronto, Canada; h School of Information, The University of Texas at Austin, Austin, Texas

**Figure:**
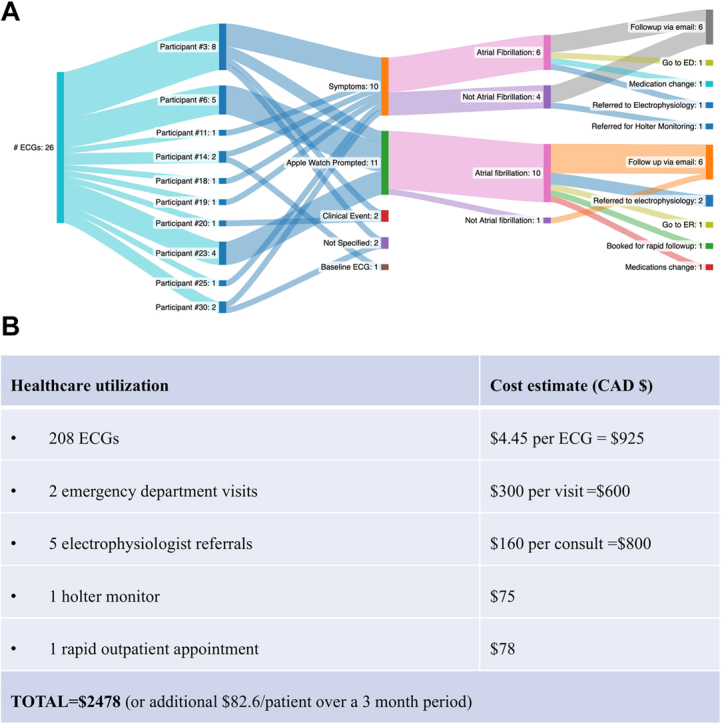

Patients with heart failure (HF) have a significant burden of atrial fibrillation (AF), an important determinant of prognosis.^1^ Given the escalating global burden of HF, it is crucial to evaluate tools for AF monitoring in HF patients.^2^ The Apple Watch (AW), a wearable device with single-lead electrocardiogram (ECG) capability, can detect AF in healthy individuals. This pilot study assessed the AW feasibility in monitoring HF patients at higher risk of AF development.

Patients at a quaternary hospital HF clinic were approached if symptomatic palpitations, post electrical cardioversion, or AF ablation. Exclusion criteria included chronic or persistent AF. Over the 3-month study period, participants emailed AW-generated 1-lead ECGs when they experienced symptoms or received an irregular pulse notification from the AW to a shared inbox. One of 3 study cardiologists reviewed each ECG within 48 hours and provided an email response on their assessment and recommendations. All ECGs were subsequently adjudicated by a blinded panel of 4 cardiologists whose consensus served as the comparator to the AW-generated interpretation in performance analysis. Statistical analyses were performed using R (version, 4.3.1). Concordance between AW interpretation and physician interpretation was assessed with Cohen’s kappa. Data collection occurred at the time of study enrollment and included patient demographics, questionnaires, and electronic medical record review. Post-study, patients completed the System Usability Scale (SUS)-12 questionnaire to assess AW usability.^3^ Ethics approval was obtained from University Health Network REB panel (19-6070).

A total of 30 participants were included between October 2020 and April 2022 with a median age of 65.5 (IQR: 54.5-73) years. Of 30 patients, 17 (57%) participants were male, 18 (60%) had HF with preserved ejection fraction, and 26 (87%) were Caucasian. 22/30 (73%) patients had a history of paroxysmal AF. 208 total AW 1-lead ECGs were sent (median 3, IQR 7 ECGs per participant). The most common reason for sending an ECG was that the AW prompted the patient for irregular heart rhythm (72/208, 35%), followed by patient experienced symptoms (52/208, 25%). Of the 208 1-lead ECGs, 69 sinus rhythm, 67 AF, 27 inconclusive, 2 “rapid heart rate,” and 43 were uninterpretable due to high heart rates. Comparing the AW readings with the expert panel's consensus, the agreement (kappa) was 0.52 (*P* < 0.05).

**Figure 1:**
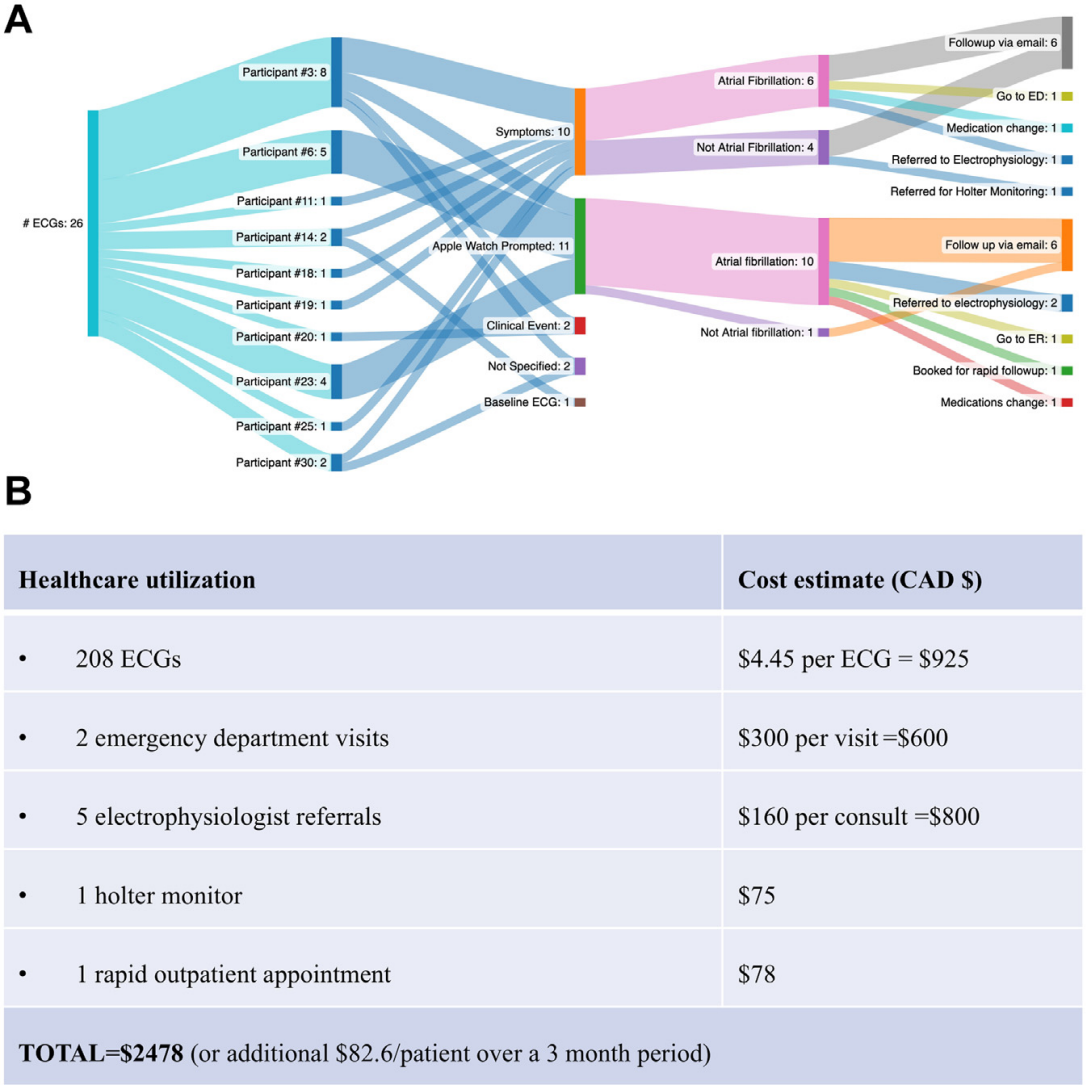
ECGs Resulting in Healthcare Utilization and the Cost Estimate of Healthcare Utilization (A) Flow diagram of end health care utilization from Apple watch electrocardiogram (ECG); (B) Breakdown of estimated costs incurrent on the health care system resulting from Apple watch ECGs.

Of the 208 ECGs, additional health care was sought in 26 cases further outlined in Figure 1A. Estimated costs incurred on the health care system when using current regional fees totaled to $2478 or average $82.6 per patient over the 3-month study period (Figure 1B).^4^

The SUS-12 results indicate that while the study population found the system “easy to use” (median score 6 out of 7 on SUS-12 question 2), they also felt the system was “cumbersome to use” (median score 6 out of 7 on SUS-12 question 8).^3^ Pearson correlation coefficient analysis revealed a positive user experience association with younger age (r = 0.87; *P* < 0.0005), higher education (r = 0.65; *P* < 0.005), and greater household income (r = 0.65; *P* < 0.005).

In an HF population at high risk of AF, all 30 participants successfully transmitted a 1-lead ECG to a shared inbox. The adherence rate was 70% (21/30 participants sent more than 1 ECG). Three cardiologists were able to promptly reply to all emails sent within 48 hours. The AW 1-lead ECG had substantial concordance for AF identification when compared to an expert cardiologist panel interpretation. Twenty-six ECGs led to health care interventions, with an average health care cost of $82.6 per patient over 3 months. A 2020 study estimated routine HF care cost to be $7,100/patient per year in Canada, thus the costs incurred on the health care system due to AW implementation was <5% of the current estimate.^5^ Our study also introduced the possibility of emergency department visit cost savings as majority of ECGs sent due to symptoms were addressed via email alone suggesting that this may be a cost-effective means of monitoring in this patient population. Taken together, these results support feasibility for a larger randomized control trial to address whether the AW could improve HF care and reduce health care costs.

To our knowledge, this is the first study to assess the feasibility, performance, health care utilization, and patient experience of the AW 1-lead ECG in detecting AF in a HF population. Limitations include the study's single-arm design given this was intended to be a pilot feasibility study. Importantly, as with any tech-driven study, the algorithm for the AW heart rate interpretation parameters shifted during our study, initially limited to interpreting ECGs with a heart rate between 50 and 120 beats/min which later changed to between 50 and 150 beats/min, leaving 43 ECGs uninterpreted and introducing system inconsistency. Most participants (87%) were Caucasian, raising concerns about wearable accuracy across skin tones. Recruitment focused on those who could send a baseline ECG, potentially limiting generalizability given the more typical HF demographic of older age and tech unfamiliarity.

In conclusion, the AW 1-lead ECG holds promise as a valuable complement to HF care. However, it is crucial to continue refining the algorithm to enhance its accessibility for elderly, lower income, and less educated patients, thus ensuring equitable benefits across diverse user groups.
